# Supplementary material for: Porcine Respiratory Coronavirus (PRCV): Isolation and Characterization of a Variant PRCV from USA Pigs
Source: Pathogens. 2023 Aug 28;12(9):1097. doi: 10.3390/pathogens12091097 (PMC10536027; doi:10.3390/pathogens12091097)
Supplement: Supplementary file 1 [file pathogens-12-01097-s001.zip › pathogens-2515616-supplementary.pdf]

Supplementary Materials

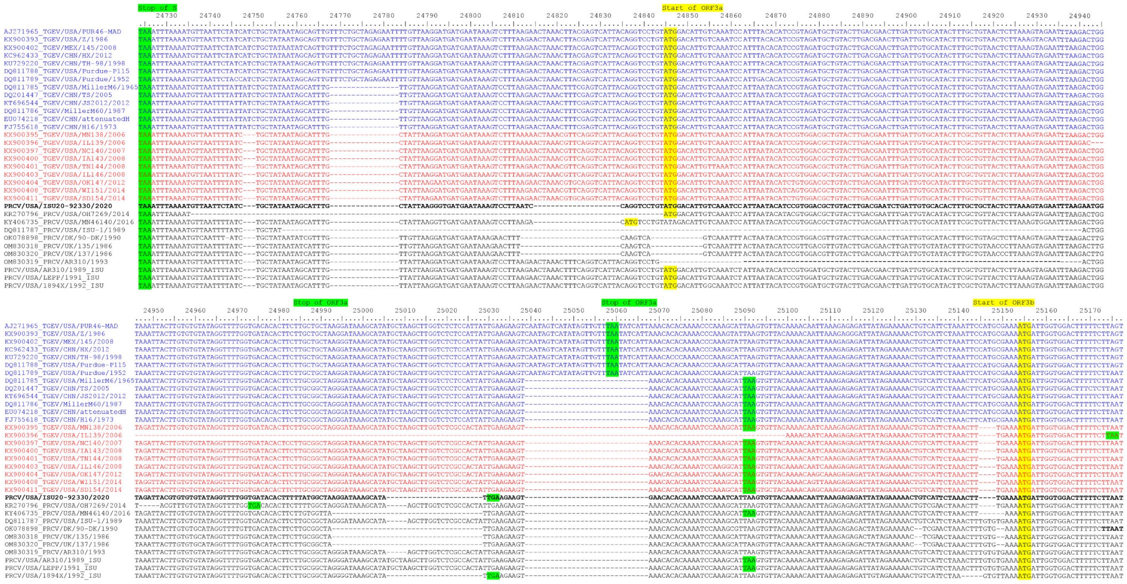

Figure S1: Comparison of the intergenic region between S and ORF3a genes, ORF3a gene, and the intergenic region between ORF3a and ORF3b genes of traditional TGEVs, variant TGEVs, and PRCVs. The representative traditional TGEVs are shown in blue color and the representative variant TGEVs are shown in red color. Nucleotides are numbered according to TGEV PUR46-MAD sequence (GenBank accession number AJ271965).

```
                                  Start of M
                                      26140     26150     26160     26170     26180     26190     26200     26210     26220     26230
                                  .|....|....|....|....|....|....|....|....|....|....|....|....|....|....|....|....|....|....|....|
AJ271965_TGEV/USA/PUR46-MAD       ATGAAGATTTTGTTAATATTAGCGTGTGTGATTGCATGCGCATGTGGAGAACGCTATTGTGCTATGAAATCCGATACAGATTTGTCATGTCGCAATAGTACA
KX900393_TGEV/USA/Z/1986          ATGAAGATTTTGTTAATATTAGCGTGTGTGATTGCATGCGCATGTGGAGAACGCTATTGTGCTATGAAATCCGATACAGATTTGTCATGTCGCAATAGTACA
KX900402_TGEV/MEX/145/2008        ATGAAGATTTTGTTAATATTAGCGTGTGTGATTGCATGCGCATGTGGAGAACGCTATTGTGCTATGAAATCCGATACAGATTTGTCATGTCGCAATAGTACA
KC962433_TGEV/CHN/HX/2012         ATGAAGATTTTGTTAATATTAGCGTGTGTGATTGCATGCGCATGTGGAGAACGCTATTGTGCTATGAAATCCGATACAGATTTGTCATGTCGCAATAGTACA
KU729220_TGEV/CHN/TH-98/1998      ATGAAGATTTTGTTAATATTAGCGTGTGTGATTGCATGCGCATGTGGAGAACGCTATTGTGCTATGAAATCCGATACAGATTTGTCATGTCGCAATAGTACA
DQ811788_TGEV/USA/Purdue-P115     ATGAAGATTTTGTTAATATTAGCGTGTGTGATTGCATGCGCATGTGGAGAACGCTATTGTGCTATGAAATCCGATACAGATTTGTCATGTCGCAATAGTACA
DQ811789_TGEV/USA/Purdue/1952     ATGAAGATTTTGTTAATATTAGCGTGTGTGATTGCATGCGCATGTGGAGAACGCTATTGTGCTATGAAATCCGATACAGATTTGTCATGTCGCAATAGTACA
DQ811785_TGEV/USA/MillerM6/1965   ATGAAGATTTTGTTGATATTAGCGTGTGTGATTGCATGCGCATGTGGAGAACGCTATTGTGCTATGAAAGACGATACAGGTTTGTCATGTCGCAATAGCACA
DQ201447_TGEV/CHN/TS/2005         ATGAAGATTTTGTTGATATTAGCGTGTGTGATTGCATGCGCATGTGGAGAACGCTATTGTGCTATGAAAGACGATACAGGTTTGTCATGTCGCAATAGCACA
KT696544_TGEV/CHN/JS2012/2012     ATGAAGATTTTGTTGATATTAGCGTGTGTGATTGCATGCGCATGTGGAGAACGCTATTGTGCTATGAAAGACGATACAGGTTTGTCATGTCGCAATAGCACA
DQ811786_TGEV/MillerM60/1987      ATGAAGATTTTGTTGATATTAGCGTGTGTGATTGCATGCGCATGTGGAGAACGCTATTGTGCTATGAAAGACGATACAGGTTTGTCATGTCGCAATAGCACA
EU074218_TGEV/CHN/attenuatedH     ATGAAGATTTTGTTGATATTAGCGTGTGTGATTGCATGCGCATGTGGAGAACGCTATTGTGCTATGAAAGACGATACAGGTTTGTCATGTCGCAATAGCACA
FJ755618_TGEV/CHN/H16/1973        ATGAAGATTTTGTTGATATTAGCGTGTGTGATTGCATGCGCATGTGGAGAACGCTATTGTGCTATGAAAGACGATACAGGTTTGTCATGTCGCAATAGCACA
KX900395_TGEV/USA/MN138/2006      ATGAAGATTTTGTTGATATTGGCGTGTGCGATTGCATGCGCATGTGGAGAACGCTATTGTGCTATGCAAGAC---ACAGGTTTGTCATGTCGCAATAGCACA
KX900396_TGEV/USA/IL139/2006      ATGAAGATTTTGTTGATATTGGCGTGTGCGATTGCATGCGCATGTGGAGAACGCTATTGTGCTATGCAAGAC---ACAGGTTTGTCATGTCGCAATAGCACA
KX900397_TGEV/USA/NC140/2007      ATGAAGATTTTGTTGATATTGGCGTGTGCGATTGTATGCGCATGTGGAGAACGCTATTGTGCTATGCAAGAC---ACAGGTTTGTCATGTCGCAATAGCACA
KX900400_TGEV/USA/IA143/2008      ATGAAGATTTTGTTGATATTGGCGTGTGCGATTGCATGCGCATGTGGAGAACGCTATTGTGCTATGCAAGAC---ACAGGTTTGTCATGTCGCAATAGCACA
KX900401_TGEV/USA/TN144/2008      ATGAAGATTTTGTTGATATTGGCGTGTGCGATTGCATGCGCATGTGGAGAACGCTATTGTGCTATGCAAGAC---ACAGGTTTGTCATGTCGCAATAGCACA
KX900403_TGEV/USA/IL146/2008      ATGAAGATTTTGTTGATATTGGCGTGTGCGATTGCATGCGCATGTGGAGAACGCTATTGTGCTATGCAAGAC---ACAGGTTTGTCATGTCGCAATAGCACA
KX900404_TGEV/USA/OK147/2012      ATGAAGATTTTGTTGATATTGGCGTGTGCGATTGCATGCGCATGTGGAGAACGCTATTGTGCTATGCAAGAC---ACAGGTTTGTCATGTCGCAATAGCACA
KX900408_TGEV/USA/WI151/2014      ATGAAGATTTTGTTAATATTGGCGTGTGCGATGGCATGCGCATGTGGGGAACGCTATTGTGCTATGCAAGAC---ACAGGTTTGTCATGTCGCAATAGCACA
KX900411_TGEV/USA/SD154/2014      ATGAAGATTTTGTTGATATTGGCGTGTGCGATTGCATGCGCATGTGGAGAACGCTATTGTGCTATGCAAGAC---ACAGGTTTGTCATGTCGCAATAACACA
PRCV/USA/ISU20-92330/2020         ATGAAGATTTTGTTGATATTGGCGTGTGCGATTGCATGCGCATGTGGAGAACGCTATTGTGCTATGCAAGAC---ACAGGTTTGTCATGTCGCAATAGCACA
KR270796_PRCV/USA/OH7269/2014     ATGAAGATTTTGTTGATATTGGCGTGTGCGCTTGCATGCGCATGTGGAGAACGCTATTGTGCTATGCAATAC---ACAGGTTTGTCATGTCGCAATAGCACA
KY406735_PRCV/USA/MN46140/2016    ATGAAGGTTTTGTTGATATTGGCGTGTGTGATTGCATGCGCATGTGGAGAACGCTATTGTGCTATGCAAGAC---ACAGGTTTTTCATGTCGCAATAGCACA
DQ811787_PRCV/USA/ISU-1/1989      ATGAAGATTTTGTTGATATTAGCGTGTGCGATTGCATGCGCATGTGGAGAACGCTATTGTGCTATGCAAGAA---ACAGGTTTGTCATGTCGCAATAGCACA
OK078898_PRCV/DK/90-DK/1990       ATGAAGATTTTGTTGATATTAGCGTGTGCGATTGCATGCACATGTGGAGAACGCTATTGTGCTATGAAAGACGATACAGGTTTGTCATGTCGCAATGGCACA
OM830318_PRCV/UK/135/1986         ATGAAGATTTTGTTGATATTAGCGTGTGCGATTGCATGCACATGTGGAGAACGCTATTGTGCTATGAAAGACGATACAGGTTTGTCATGTCGCAATGGCACA
OM830320_PRCV/UK/137/1986         ATGAAGATTTTGTTGATATTAGCGTGTGCGATTGCATGCACATGTGGAGAACGCTATTGTGCTATGAAAGACGATACAGGTTTGTCATGTCGCAATGGCACA
OM830319_PRCV/AR310/1993          ATGAAGATTTTGTTGATATTAGCGTGTGCGATTGCATGCGCATGTGGAGAACGCTATTGTGCTATGCAAGAA---ACAGGTTTGTCATGTCGCAATAGCACA
PRCV/USA/AR310/1989_ISU           ATGAAGATTTTGTTGATATTAGCGTGTGCGATTGCATGCGCATGTGGAGAACGCTATTGTGCTATGCAAGAA---ACAGGTTTGTCATGTCGCAATAGCACA
PRCV/USA/LEPP/1991_ISU            ATGAAGATTTTGTTGATATTAGCGTGTGCGATTGCATGCGCATGTGGAGAACGCTATTGTGCTATGCAAGAA---ACAGGTTTGTCATGTCGCAATAGCACA
PRCV/USA/1894X/1992_ISU           ATGAAGATTTTGTTGATATTAGCGTGTGCGATTGTATGCGCATGTGGAGAACGCTATTGTGCTATGCAAGAA-----GGTTTGTCATGTCGCAATAGCACA

                                     26240     26250     26260     26270     26280     26290     26300     26310     26320     26330
                                  ....|....|....|....|....|....|....|....|....|....|....|....|....|....|....|....|....|....|....|....|
AJ271965_TGEV/USA/PUR46-MAD       GCGTCTGATTGTGAGTCATGCTTCAACGGAGGCGATCTTATTTGGCATCTTGCAAACTGGAACTTCAGCTGGTCTATAATATTGATCGTTTT------TA
KX900393_TGEV/USA/Z/1986          GCGTCTGATTGTGAGTCATGCTTCAACGGAGGCGATCTTATTTGGCATCTTGCAAACTGGAACTTCAGCTGGTCTATAATATTGATCGTTTT------TA
KX900402_TGEV/MEX/145/2008        GCGTCTGATTGTGAGTCATGCTTCAACGGAGGCGATCTTATTTGGCATCTTGCAAACTGGAACTTCAGCTGGTCTATAATATTGATCGTTTT------TA
KC962433_TGEV/CHN/HX/2012         GCGTCTGATTGTGAGTCATGCTTCAACGGAGGCGATCTTATTTGGCATCTTGCAAACTGGAACTTCAGCTGGTCTATAATATTGATCGTTTT------TA
KU729220_TGEV/CHN/TH-98/1998      GCGTCTGATTGTGAGTCATGCTTCAACGGAGGCGATCTTATTTGGCATCTTGCAAACTGGAACTTCAGCTGGTCTATAATATTGATCGTTTT------TA
DQ811788_TGEV/USA/Purdue-P115     GCGTCTGATTGTGAGTCATGCTTCAACGGAGGCGATCTTATTTGGCATCTTGCAAACTGGAACTTCAGCTGGTCTATAATATTGATCGTTTT------TA
DQ811789_TGEV/USA/Purdue/1952     GCGTCTGATTGTGAGTCATGCTTCAACGGAGGCGATCTTATTTGGCATCTTGCAAACTGGAACTTCAGCTGGTCTATAATATTGATCGTTTT------TA
DQ811785_TGEV/USA/MillerM6/1965   GCGTCTGATTGTGAGTCATGCTTCAACGGAGGCGATCTTATTTGGCATCTTGCAAACTGGAACTTCAGCTGGTCTATAATATTGATTATTTT------TA
DQ201447_TGEV/CHN/TS/2005         GCGTCTGATTGTGAGTCATGCTTCAACGGAGGCGATCTTATTTGGCATCTTGCAAACTGGAACTTCAGCTGGTCTATAATATTGATTATTTT------TA
KT696544_TGEV/CHN/JS2012/2012     GCGTCTGATTGTGAGTCATGCTTCAACGGAGGCGATCTTATTTGGCATCTTGCAAACTGGAACTTCAGCTGGTCTATAATATTGATTATTTT------TA
DQ811786_TGEV/MillerM60/1987      GCGTCTGATTGTGAGTCATGCTTCAACGGAGGCGATCTTATTTGGCATCTTGCAAACTGGAACTTCAGCTGGTCTATAATATTGATTATTTTTATTTTTA
EU074218_TGEV/CHN/attenuatedH     GCGTCTGATTGTGAGTCATGCTTCAACGGAGGCTATCTTATTTGGCATCTTGCAAACTGGAACTTCAGCTGGTCTATAATATTGATTATTTT------TA
FJ755618_TGEV/CHN/H16/1973        GCGTCTGATTGTGAGTCATGCTTCAACGGAGGCTATCTTATTTGGCATCTTGCAAACTGGAACTTCAGCTGGTCTATAATATTGATTATTTT------TA
KX900395_TGEV/USA/MN138/2006      GTGTCTGATTGTGAGTCATGCTTCAACGGGGGCGATCTTATTTGGCATCTTGCAAACTGGAACTTCAGCTGGTCTATAATATTGATCATTTT------TA
KX900396_TGEV/USA/IL139/2006      GTGTCTGATTGTGAGTCATGCTTCAACGGGGGCGATCTTATTTGGCATCTTGCAAACTGGAACTTCAGCTGGTCTATAATATTGATCATTTT------TA
KX900397_TGEV/USA/NC140/2007      GTGTCTGATTGTGAGTCATGCTTCAACGGGGGCGATCTTATTTGGCATCTTGCAAACTGGAACTTCAGCTGGTCTATAATATTGATCATTTT------TA
KX900400_TGEV/USA/IA143/2008      GTGTCTGATTGTGAGTCATGCTTCAACGGGGGCGATCTTATTTGGCATCTTGCAAACTGGAACTTCAGCTGGTCTATAATATTGATCATTTT------TA
KX900401_TGEV/USA/TN144/2008      GTGTCTGATTGTGAGTCATGCTTCAACGGGGGCGATCTTATTTGGCATCTTGCAAACTGGAACTTCAGCTGGTCTATAATATTGATCATTTT------TA
KX900403_TGEV/USA/IL146/2008      GTGTCTGATTGTGAGTCATGCTTCAACGGGGGCGATCTTATTTGGCATCTTGCAAACTGGAACTTCAGCTGGTCTATAATATTGATCATTTT------TA
KX900404_TGEV/USA/OK147/2012      GTGTCTGATTGTGAGTCATGCTTCAACGGGGGCGATCTTATTTGGCATCTTGCAAACTGGAACTTCAGCTGGTCTATAATATTGATCATTTT------TA
KX900408_TGEV/USA/WI151/2014      GTGTCTGATTGTGAGTCATGCTTCAACGGAGGCGATCTTATTTGGCATCTTGCAAACTGGAACTTCAGCTGGTCTATAATATTGATCATTTT------TA
KX900411_TGEV/USA/SD154/2014      GTGTCTGAGTGTGAGTCATGCTTCAACGGGGGCGATCTTATTTGGCATCTTGCAAACTGGAACTTCAGCTGGTCTATAATATTGATCATTTT------TA
PRCV/USA/ISU20-92330/2020         GTGTCTGATTGTGAGTCATGCTTCAACGTAGGCGATCTTATTTGGCATCTTGCAAACTGGAACTTCAGCTGGTCTATAATATTGATCATTTT------TA
KR270796_PRCV/USA/OH7269/2014     GTGTCTGATTGTGAGTTATGCTTCAACGGAGGCGATCTTATATGGCATCTTGCAAACTGGAACTTCAGCTGGTCTATAATATTGATCATTTT------TA
KY406735_PRCV/USA/MN46140/2016    GTGTCTGATTGTGAGTTATGCTTCAACGGAGGCGATCTTATTTGGCATCTTGCAAACTGGAACTTCAGCTGGTCTATAATATTGATCATTTT------TA
DQ811787_PRCV/USA/ISU-1/1989      GTGTCTGATTGTGAGTCATGCTTCAACGGAGGCGATCTTATTTGGCATCTTGCAAACTGGAACTTCAGCTGGTCTATAATATTGATCATTTT------TA
OK078898_PRCV/DK/90-DK/1990       GCGTCTGATTGTGAGTCATGCTTCAACAGAGGCGATCTTATTTGGCATCTTGCAAACTGGAACTTCAGCTGGTCTATAATATTGATCATTTT------TA
OM830318_PRCV/UK/135/1986         GCGTCTGATTGTGAGTCATGCTTCAACAGAGGCGATCTTATTTGGCATCTTGCAAACTGGAACTTCAGCTGGTCTATAATATTGATCATTTT------TA
OM830320_PRCV/UK/137/1986         GCGTCTGATTGTGAGTCATGCTTCAACAGAGGCGATCTTATTTGGCATCTTGCAAACTGGAACTTCAGCTGGTCTATAATATTGATCATTTT------TA
OM830319_PRCV/AR310/1993          GTGTCTGATTGTGAGTCATGCTTCAACGGAGGCGATCTTATTTGGCATCTTGCAAACTGGAACTTCAGCTGGTCTATAATATTGATCATTTT------TA
PRCV/USA/AR310/1989_ISU           GTGTCTGATTGTGAGTCATGCTTCAACGGAGGCGATCTTATTTGGCATCTTGCAAACTGGAACTTCAGCTGGTCTATAATATTGATCATTTT------TA
PRCV/USA/LEPP/1991_ISU            GTGTCTGATTGTGAGTCATGCTTCAACGGAGGCGATCTTATTTGGCATCTTGCAAACTGGAACTTCAGCTGGTCTATAATATTGATCATTTT------TA
PRCV/USA/1894X/1992_ISU           TCGTCTGAGTGTGAGTCATGCTTCAACGGAGGCGATCTTGTTTGGCATCTTGCAAACTGGAACTTCAGCTGGTCTATAATATTGATCATTTT------TA
```

Figure S2. Comparison of M gene of traditional TGEVs, variant TGEVs, and PRCVs. The representative traditional TGEVs are shown in blue color and the representative variant TGEVs are shown in red color. Nucleotides are numbered according to TGEV PUR46-MAD sequence (GenBank accession number AJ271965).
